# Biomechanics of the retina: experimental and clinical perspectives

**DOI:** 10.1186/s12938-026-01599-2

**Published:** 2026-07-07

**Authors:** Brianna E. Samson, Mohamed H. Nashnoush, Corey A. Smith

**Affiliations:** 1 School of Biomedical Engineering, Dalhousie University, Halifax, NS Canada; 2 Faculty of Medicine, Dalhousie University, Halifax, NS Canada; 3 Department of Ophthalmology and Visual Sciences, Dalhousie University, 1276 South Park Street, Victoria Building, Room 2035, Halifax, NS B3H 2Y9 Canada

**Keywords:** Retina, Tissue models, Computational modeling, Clinical relevance

## Abstract

The retina is an essential component of the visual system, a delicate neural tissue that converts light signals into electrical impulses and links the visual environment to the brain. Its structure and functional performance depend on multiple layers of interconnected cells and extracellular matrix components that together provide mechanical support. The retina’s biomechanical properties, including elasticity, stiffness, and tensile strength, govern how it deforms and recovers under physiological load and mechanical stress. Quantifying these properties is essential for understanding how the retina resists tractional and compressive forces that can lead to injury or disease. Techniques including tensile testing, atomic force microscopy, optical coherence elastography, Brillouin microscopy, and computational modeling have advanced our understanding of retinal stiffness and revealed altered tissue mechanics in ocular disease. This review describes current knowledge of retinal structure and material behavior, experimental and computational approaches used to assess mechanical properties, and how biomechanical changes contribute to the development and progression of retinal disorders.

**Supplementary Information:**

The online version contains supplementary material available at https://doi.org/10.1186/s12938-026-01599-2.

## Introduction

Biomechanics within the eye play an essential role in both the structural integrity and visual function. Although the cornea and sclera are recognized as load-bearing tissues, retinal biomechanics are less well-defined [1]. As an integral part of the central nervous system [2], retinal function depends on the coordinated mechanical interaction between the cellular layers and the extracellular matrix (ECM).

**Fig. 1:**
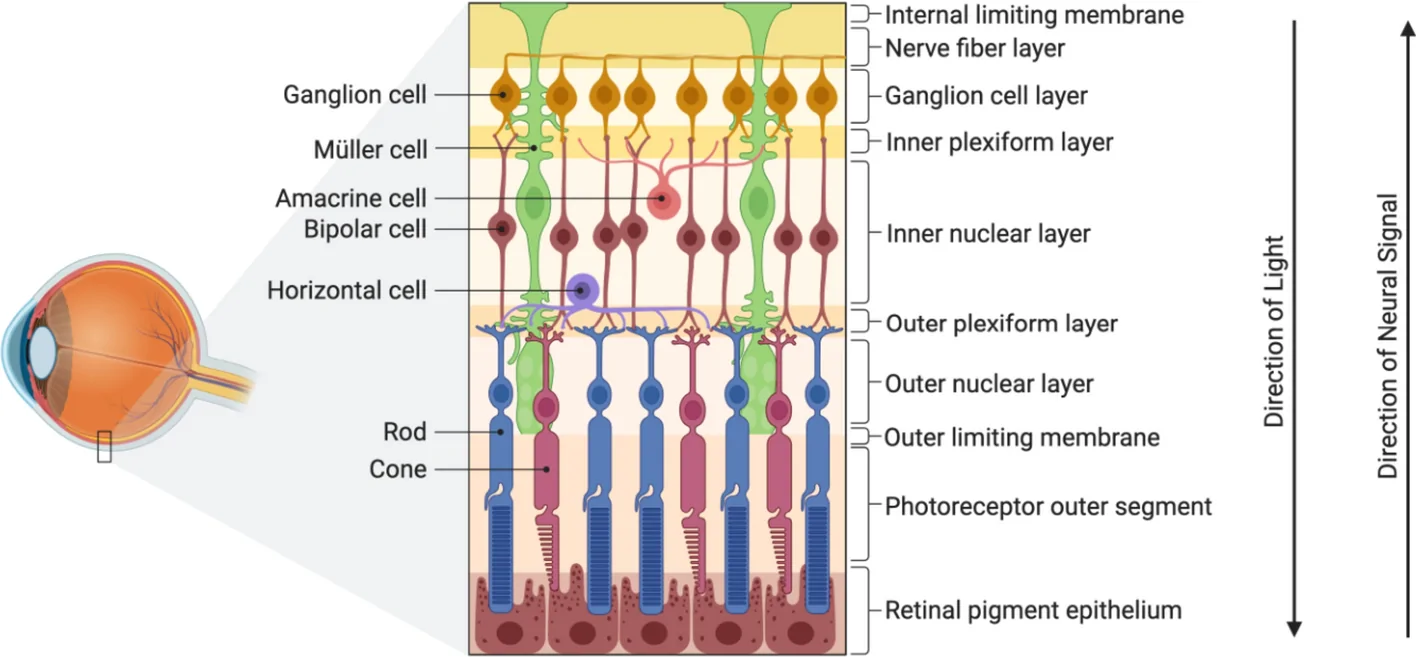
Structural organization of the human retina showing major neural and glial cells and corresponding anatomical layers. Arrows indicate the direction of incoming light and the opposing propagation of neural signals. Created in BioRender. (Samson, B. (2026) https://BioRender.com/v9p0u4c)

The retina is a layered structure that is defined by a specific cellular composition: retinal pigment epithelium (RPE) cells, photoreceptors, bipolar cells, ganglion cells, horizontal cells, amacrine cells, and Müller cells (Fig. 1). At the outermost layer, RPE cells form a supportive monolayer below the photoreceptors, nourishing the neural retina through nutrient and waste diffusion [3]. Photoreceptors, specifically rods and cones, are photosensitive neural cells composed of inner and outer segments that function to capture and transduce light into electrochemical signals. Bipolar cells act as interneurons that connect and relay signals from photoreceptors to ganglion and amacrine cells [4]. Finally, the ganglion cells function as bridging neurons, receiving visual information from downstream neurons and transmitting those signals through the optic nerve to the visual centers in the brain [5, 6]. Regulation of neuronal synapses through feedback and feedforward signaling is the role of horizontal cells (between photoreceptors and bipolar cells) and amacrine cells (between bipolar cells and ganglion cells) [7]. The glial cells of the retina, astrocytes, microglia and Müller cells, are in close contact with all retinal cells, functioning as mechanosensors and ECM modulators [8].

Importantly, retinal biomechanics emerge not only from the intrinsic mechanical properties of individual cellular components, but also from the collective interactions between retinal cells and the surrounding ECM. These interactions give rise to non-linear, anisotropic, and viscoelastic tissue behavior. Biomechanical homeostasis is, therefore, essential for preserving retinal lamination, maintaining cellular connectivity, and supporting tissue resilience under physiological loading conditions. However, the retina is constantly subjected to forces such as intraocular pressure (IOP) and dynamic tractional forces from neighboring structures. The dysregulation of these forces can contribute to vision-threatening conditions, including retinal detachment (RD), tractional macular disorders, diabetes and age-related macular degeneration (AMD). For instance, static and dynamic tractions can overwhelm retinal adhesion, leading to the formation of tears and detachment. Similarly, injury-induced ECM remodeling, and cellular hypertrophy can alter tissue mechanics, promote fibrosis, and contribute to visual impairment.

Despite the growing recognition of retinal biomechanics in health and disease, accurately characterizing retinal mechanical properties remains experimentally challenging. The retina is a fragile and heterogenous tissue composed of micrometer-scale layers that are sensitive to experimental conditions. As a result, the current literature demonstrates substantial variability in reported mechanical properties. Direct comparison between studies is often difficult due to differences in species, testing methodology, hydration state, temperature, retinal region, and sample preparation. Furthermore, inconsistencies in terminology, experimental protocols, and analytical models have limited the development of standardized frameworks for interpreting retinal mechanical behavior across studies.

This review will use a bioengineering perspective to examine the mechanical behavior of the retina, with particular emphasis on the contributions of retinal neurons, glial cells, ECM components, and intraocular forces to tissue integrity and mechanical homeostasis. In addition, this review evaluates current experimental and emerging methodologies used to characterize the retina while highlighting the relevance of biomechanical dysregulation in retinal pathology.

To identify literature relevant to the biomechanics and mechanical characterization of the retina, a systematic search was conducted in January 2025 using three electronic databases: PubMed, Embase, and Google Scholar. The following keywords and truncated terms were used: *retina*; biomechanic*; mechanic*; *Müller*; *tensile*; *tension*; *traction*; *extracellular matrix*; *optical coherence elastography*; *atomic force microscopy*; *Brillouin microscopy*; *modulus*; *stiffness*; *elasticity*; *diabetes*; *detachment*; *myopia*. Boolean operators (AND, OR) were applied to combine terms, and search strings were iteratively refined based on database-specific indexing conventions and relevance to the review objectives. No formal date restriction was applied; however, priority was given to studies published within the last two decades, with seminal earlier works included where appropriate. Inclusion criteria encompassed peer-reviewed original research articles, review articles, and book chapters reporting on retinal biomechanical properties, experimental characterization methods, or clinically relevant mechanical phenomena. Conference abstracts, non-peer-reviewed sources, and studies not available in English were excluded. Additional relevant studies were identified through manual screening of the reference lists.

## Measuring retinal biomechanical properties

Characterizing retinal biomechanics requires experimental techniques to measure tissue deformation in response to a controlled applied load [1]. The principal approaches include mechanical tests and optical-based methods, such as uniaxial tensile testing, atomic force microscopy (AFM), optical coherence elastography (OCE), and Brillouin microscopy. These techniques not only rely on different underlying physical principles, but also differ in applicability, measurable parameters (e.g., elasticity, stiffness, viscoelasticity), and potential for clinical translation (Supplementary Table 2). The terms “stiffness” and “elasticity” are frequently used interchangeably in the literature; however, they represent distinct concepts. Elasticity refers to the ability of tissue to deform and return to its original shape, often quantified as the elastic modulus; in contrast, stiffness reflects the tissue’s structural resistance to deformation and depends on both material properties and tissue geometry. Tissue stiffness and elasticity are important to the retina, because both play a role in structural integrity and how the tissue responds to mechanical stress.

### Tensile testing

**Fig. 2:**
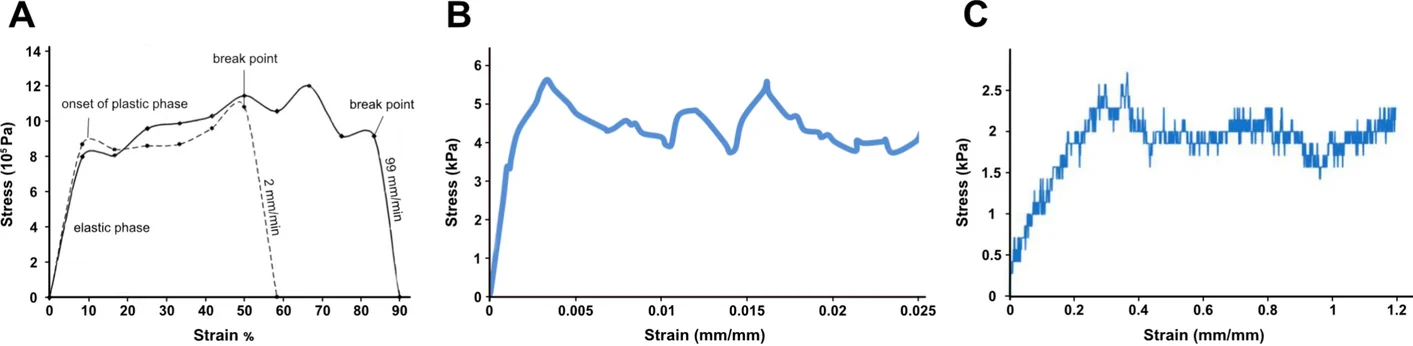
Stress–strain behavior of porcine retinal tissue under uniaxial tension. The curves show an initial linear elastic region followed by a non-linear plastic region. **A** Porcine retinal tissue clamped horizontally and stretched at a velocity of 2 mm/min (0.033 m/s) and 99 mm/min (1.65 mm/s), using the MINIMAT biomaterial tester. Young’s modulus: 100 kPa and 110 kPa, respectively. Figure adapted from Wollensak and Spoerl [11]. **B** Porcine retinal tissue tested in air at room temperature at a velocity of 0.88%/s, using the TC-3F bioreactor. Elastic modulus: 2870 kPa. Figure adapted from Belgio et al. [13]. **C** Porcine retinal tissue tested in air at room temperature at a velocity of 0.1 mm/s, using the Bose Endura TEC ELF 3200 system. Elastic modulus: 13.4 kPa. Figure adapted from Belgio et al. [13]

Uniaxial tensile testing characterizes retinal mechanics by stretching excised strips under controlled load, recording force and elongation [1, 9, 10]. As with other soft tissues, the retina exhibits non-linear, viscoelastic mechanical behavior; however, the shapes of retinal stress–strain curves vary substantially across studies. Some studies describe a biphasic response consisting of an initial linear elastic region followed by an extended non-linear region prior to failure (Fig. 2). In these studies, the retina initially demonstrates a relatively proportional stress–strain relationship before transitioning to irreversible deformation at higher strains. Wollensak and Spoerl further demonstrated that increasing loading velocity from 2 mm/min (0.033 m/s) to 99 mm/min (1.65 mm/s) shortened the non-linear deformation region (Fig. 2) and increased the stress at failure, consistent with rate-dependent viscoelastic behavior [11]. Similarly, Belgio et al. observed narrow initial linear regions followed by progressive non-linear deformation before rupture. This behavior may reflect the accumulation of microstructural damage, suggesting that the retina is capable of absorbing substantial strain prior to mechanical failure [10–12].

**Fig. 3:**
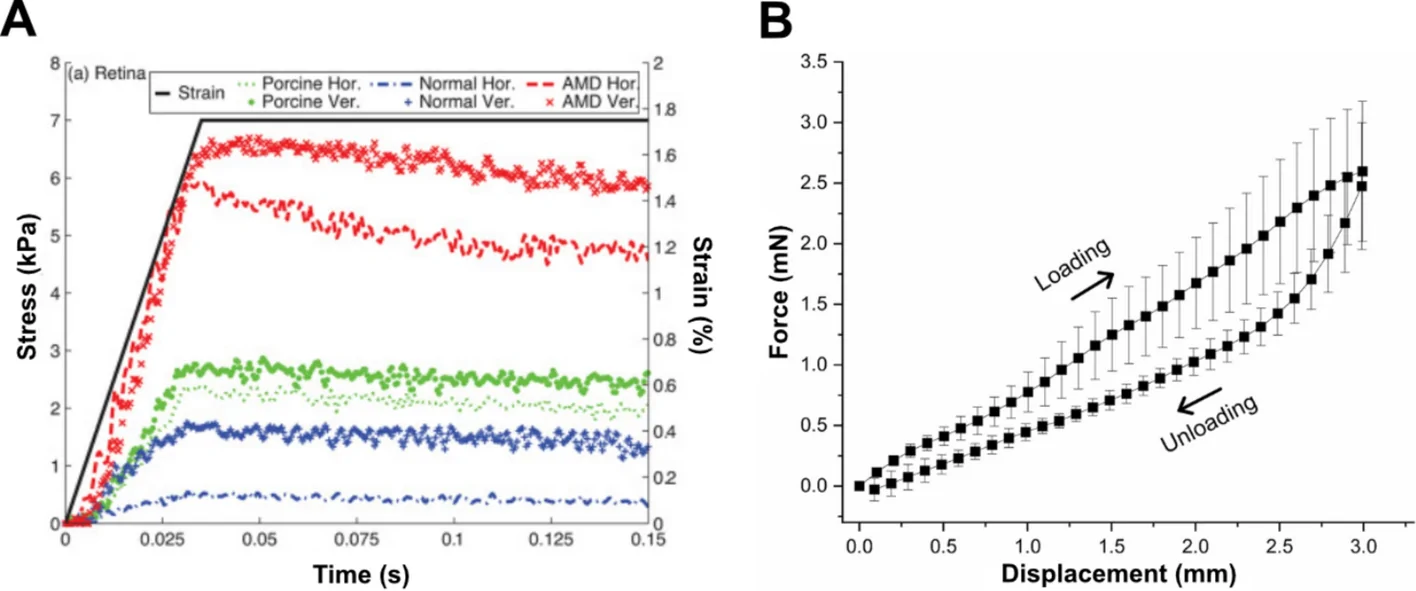
Viscoelastic and anisotropic mechanical behavior of retinal tissue. **A** Stress-relaxation curve of human and porcine retinal tissue under uniaxial tension. Under constant strain, stress decreases over time, consistent with viscoelastic behavior. The rate of stress relaxation differs between specimens tested along the superior–inferior (vertical) and nasal-temporal (horizontal) axes, indicating mechanical anisotropy in the plane of the retina. Figure adapted from Zhang et al. [18]. **B** Force–displacement curve from a loading–unloading cycle in a multiaxial small-punch test of porcine retinal tissue. The presence of a hysteresis loop indicates energy dissipation between loading and unloading phases, reflecting viscous behavior. Figure reprinted from Belgio et al. [10]

Conversely, other studies demonstrate a J-shaped stress–strain response characterized by an initially non-linear toe region at low strains, followed by a progressively more linear relationship at higher strains [9, 14–16]. Chen et al. further demonstrated that this mechanical response varies with retinal orientation, vascularity, and temperature. Retinal strips containing major blood vessels exhibited greater stress response (steeper slope) than avascular regions, while differences between the circumferential and axial orientation of blood vessels, with respect to the optic nerve, demonstrated mechanical anisotropy (Fig. 3) [15]. Temperature-dependent differences have also been observed, with retinal tissue tested at lower temperatures exhibiting a higher transition modulus compared to physiological conditions [15]. This behavior likely reflects the gradual recruitment and alignment of load-bearing extracellular and cellular retinal components, including collagenous basement membrane structures.

Reported differences in stress–strain behaviors may arise from variations in experimental methodology and sample preparation. One potential contributing factor is force transducer sensitivity. Belgio et al. (Fig. 2) used higher-capacity load cells (~ 4.4 N and 22 N, respectively), which may have reduced the sensitivity to subtle force changes at low strains and could, therefore, obscure the initial non-linear response of the retina [12, 13]. In contrast, studies using lower-capacity and more sensitive load cells, such as the 0.5 N load cell used by Chen et al., may better resolve mechanical behavior at low forces, making the initial non-linear response more apparent [9]. However, because load cell capacity is not consistently reported across all studies, the contribution of instrumentation cannot be fully determined.

Tensile testing has also revealed that the retina is mechanically heterogenous, with regional differences influenced by structural composition, and exhibits viscoelastic behavior. The presence of vasculature significantly increases retinal stiffness, as strips containing major blood vessels (arteries or veins) have up to 3.5 times greater transition moduli values than avascular regions [15]. The internal limiting membrane (ILM) is also a major contributor to the retina’s tensile strength with its removal resulting in a reduction of tensile strength by ~ 50% in human tissue [17]. In addition to spatial differences, under tensile load the retina exhibits viscoelastic characteristics that depend on loading rate and time. Stress-relaxation tests demonstrate a decrease in stress over time under constant strain [18], while hysteresis during loading and unloading cycles indicates energy dissipation (Fig. 3) [10]. Furthermore, experimental studies have shown that high strain rate (e.g., 99 mm/min) results in an increased Young’s modulus, increased stress at break, and a shorter plastic region of irreversible deformation [11]. Such findings could explain the retina’s heightened susceptibility to tearing following high-impact trauma [11].

Importantly, the substantial variability in reported mechanical parameters, with elastic moduli values ranging from 0.3 to 2800 kPa (Supplementary Table 1), cannot be attributed only to the non-linear nature of the stress–strain response. This broad range reflects the combined influence of species differences, anatomical region, retinal orientation, vascularity, tissue hydration, strain rate, testing environment, sample preparation, and the specific mechanical testing equipment employed [1, 10, 14]. Consequently, elastic moduli values reported across studies should not be interpreted as directly interchangeable material constants, but instead as condition-dependent estimates of retinal mechanical behavior.

**Fig. 4:**
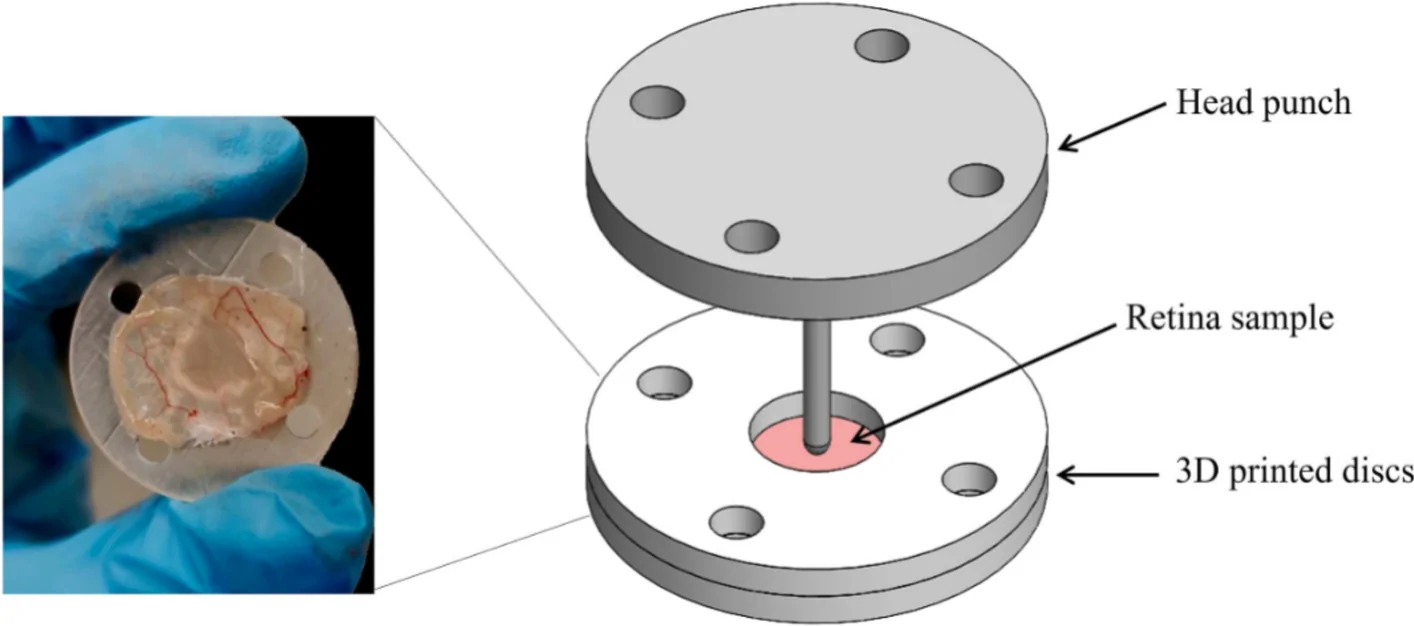
Experimental setup of the small-punch test. Custom 10 mm circular disks containing one central hole and four peripheral holes were created via stereolithography. The retinal tissue is placed central, and disks are screwed together using the peripheral holes; a hemispherical stainless-steel head punch is used to perform a loading and unloading cycle. Figure reprinted from Belgio et al. [10]

Although prevalent, uniaxial tensile testing has limitations when applied to retinal tissue. Cutting a flat strip from the retina’s naturally curved surface severs native connections and can introduce artificial stresses, such as tension on the inner surface [1]. Moreover, loading the tissue in a single axis does not fully replicate the multiaxial strains the retina experiences in vivo [1, 10]. To address these concerns, complementary techniques, such as the small-punch test, have emerged, in which a circular tissue sample is clamped at the periphery and deformed by a small indenter that presses perpendicularly into the tissue, producing biaxial stretch (Fig. 4) [10]. The small-punch test yields a load–displacement curve that can be interpreted via finite element simulations to extract the retina’s elastic properties. Using this approach on porcine retina, Belgio et al. reported an elastic modulus of 5.5 kPa under biaxial loading conditions, closely matching the 6.0 kPa modulus obtained from their uniaxial tensile experiments performed under comparable dry testing conditions, with reduced sample damage. Uniaxial tensile testing, complemented by biaxial methods, has advanced our understanding of retinal mechanics. These techniques demonstrate that the retina’s mechanical response is non-linear, viscoelastic and not defined by a single modulus value, but instead depends on region, orientation and loading conditions.

### Atomic force microscopy

AFM generates high-resolution topographical and mechanical maps of the retina by using a fine cantilever with a spherical or sharp tip to perform nanoindentation, measuring the force-distance response in the tissue [19]. Fitting the resulting curves to contact models allows for the calculation of tissue elasticity, specifically Young’s modulus, enabling quantification of tissue stiffness at the cellular and subcellular scale. The spatial resolution of AFM is governed by the contact area between the probe and the sample, typically tens to hundreds of μm^2^ at 1 μm indentation depth for spherical bead probes [20], and substantially smaller for sharp pyramidal tips.

Gutmann, Salvador [20] mapped murine retinal layers, finding Young’s modulus values ranging from 0.15 kPa in the inner nuclear layer to 0.4 kPa in the inner plexiform layer (IPL), demonstrating heterogeneity across layers. AFM has also facilitated mechanical profiling of the ILM, revealing non-uniform stiffness patterns attributed to the heterogenous distribution of neuronal somata, nerve fibers, and Müller endfeet across the surface [21]. Henrich et al. reported that the retinal stiffness of the ILM retinal surface was five times higher (224.6 kPa) than the opposing vitreal surface (44 kPa) [22].

Despite its high spatial resolution, AFM has important limitations relevant in the context of retinal biomechanics. AFM is inherently a contact-based, ex vivo technique: the cantilever probe must physically indent the tissue, requiring sample preparation and direct probe to tissue contact that is not possible to measure in vivo and may disturb the native tissue hydration of the retinal surface. Furthermore, AFM can only measure tissue surfaces or thin sections, and characterization of deeper retinal layers requires physical sectioning, which itself can introduce artifacts. Candiello et al. demonstrated that dehydration during TEM preparations of human ILM tissue (a step also involved in fixation protocols) reduces the apparent ILM thickness by 30–50% and increases apparent stiffness by at least 30% relative to AFM measurements performed under native tissue hydration [23].

### Brillouin microscopy

**Fig. 5:**
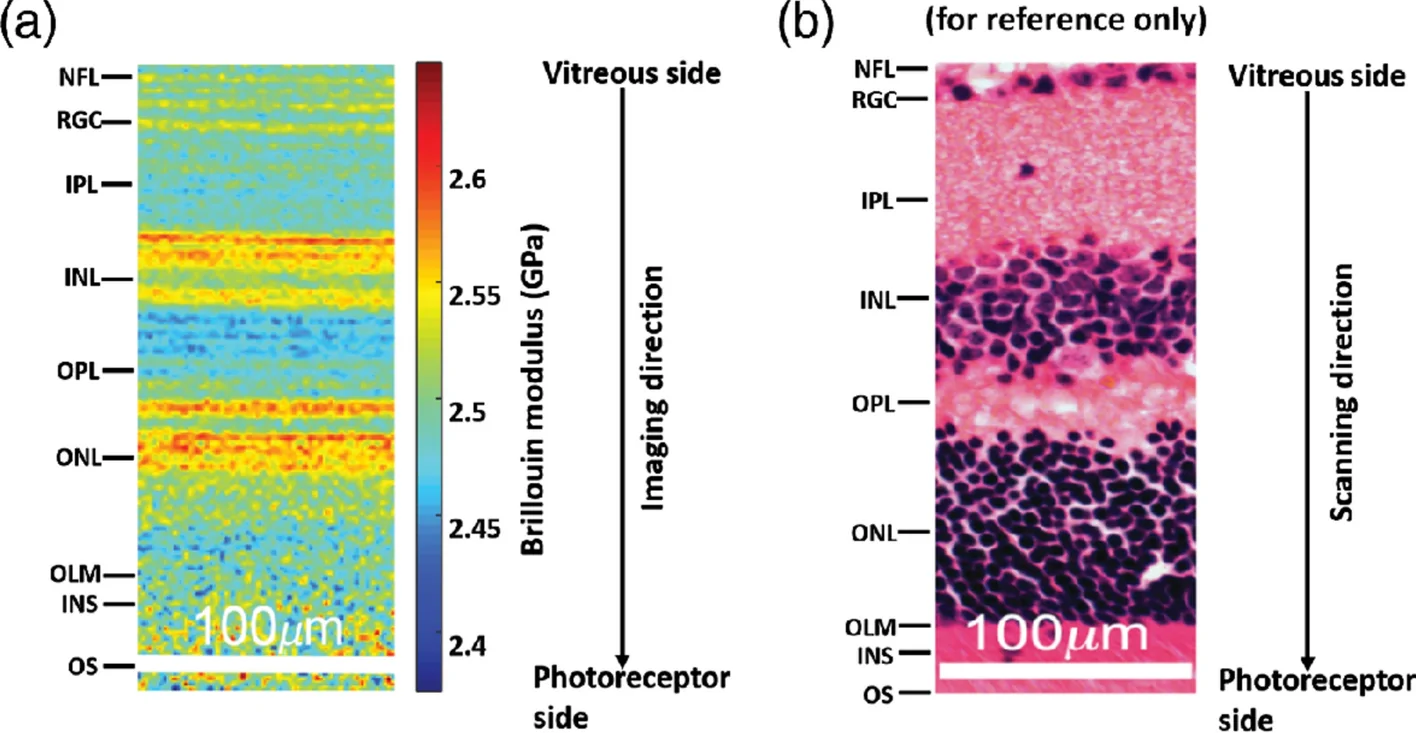
Brillouin microscopy of the mouse retina **a**) Brillouin frequency-shift map through the full thickness of the retina, **b**) histology of mouse retina (for reference only). Figure adapted from Ambekar et al. [25]

Brillouin microscopy provides a non-contact approach to map the viscoelastic properties of retinal layers by measuring frequency shifts in scattered light, caused by acoustic waves [24]. The frequency shift between incident and scattered light (Brillouin shift) is determined by the propagation velocity of these acoustic waves, which in turn depends on the tissue’s longitudinal elastic modulus and density [24]. Because it requires only a focused laser beam and no mechanical contact or exogenous contrast agent, Brillouin microscopy is inherently well-suited to imaging delicate tissues such as the retina at subcellular resolution [20, 25]. Ambekar et al. found that nuclear layers exhibited higher Brillouin modulus values than the plexiform layers, attributed to the higher density of cell bodies (Fig. 5) [25]. Brillouin microscopy has also been used to spatially resolve layer-specific increases in retinal stiffness in a mouse model of excitotoxic retinal damage, with significantly elevated Brillouin frequency shifts detected within 24 h of injury, supporting its potential as a non-contact tool for detecting early biomechanical changes in retinal disease [26].

However, the interpretation of Brillouin measurements in the context of tissue mechanics requires careful consideration. Brillouin microscopy measures the longitudinal (bulk) modulus at GHz frequencies, which is dominated by tissue water content and compressibility, whereas AFM probes the Young’s or shear modulus at quasi-static rates, capturing the equilibrium mechanics of the solid extracellular network. Consequently, the same structural change (e.g., altered cross-linking or hydration) can produce divergent apparent stiffness depending on the modality employed, and the Brillouin-derived viscoelastic modulus of the tissue cannot be equated with Young’s modulus or shear modulus values reported by AFM or OCE [20].

### Optical coherence elastography

Optical coherence elastography combines the imaging power of optical coherence tomography with elastography, enabling non-invasive, depth-resolved quantification of retinal elasticity [27–29]. In OCE, a mechanical excitation (e.g., static compression, air-pulse perturbation, or a piezoelectric actuator) is applied to the tissue, and the resulting displacement or propagating elastic wave is detected using phase-sensitive OCT. The local mechanical properties, principally tissue stiffness or Young's modulus, are then inferred from the measured displacement field or wave propagation velocity. OCE studies have consistently demonstrated a stiffness gradient across the retina: increasing from the inner ganglion cell layer to the outer photoreceptor and RPE layers [27, 30]. Li et al. measured elastic wave propagation in rabbit retinal layers, reporting elastic wave speeds of 3.66 ± 0.36 m/s in the nerve fiber layer to the IPL, 5.33 ± 0.07 m/s in the inner nuclear layer to the outer nuclear layer, and 6.85 ± 0.37 m/s in the RPE [30]. These mechanical differences correspond with compositional and functional specialization of each layer, potentially linked to cellular density and synaptic circuitry influencing local load-bearing capacity [20, 25]. OCE has also revealed regional mechanical differences, with peripheral regions generally more compliant than central regions, and has recently been used for in vivo elasticity mapping in animal models and postmortem human eyes [27, 29].

This approach can preserve normal tissue attachments and can capture dynamic responses, but faces challenges such as shallow depth of measurements, limited excitation methods to reach the retina, and simplified models that do not capture the anisotropic nature of the retina [31]. Furthermore, the stiffness values derived from OCE are highly sensitive to the frequency and amplitude of the applied excitation, meaning that measurements obtained under different loading conditions are not directly comparable. While this technique can be used for in vivo applications, careful corrections must be made for ocular motion artifacts and spatial resolution. It is important to emphasize that the moduli reported by OCE, Brillouin microscopy, and AFM are not equivalent quantities and should not be compared directly without careful qualification.

## Models for retinal biomechanics

### Tissue models

A variety of tissue models have been used to study retinal biomechanics, including animal models and human cadaver eyes. Small animals, including mice, rats, guinea pigs and chicks have been used to investigate the mechanical properties of the retina, applying AFM or compression testing to excised retinal or ILM samples [21, 24, 32, 33]. Small animal models offer practical advantages, such as genetic manipulation and greater accessibility due to short gestational and developmental timelines [34]. However, these animals differ anatomically from human eyes with a relatively small retinal surface area, lack of a cone-dense macula region, and differences in vasculature pattern (e.g., guinea pigs have an avascular retina) [35], limiting their ability to replicate human central retinal mechanics. Large animal models, most commonly porcine eyes, have been used for tensile testing [9, 10, 13–15] as they have a similar globe size to that of the human eye and a fully vascularized retina [35]. However, like small mammals, porcine lack a true macula region, and have a visual streak of cones that does not fully replicate the central retina of humans.

The interspecies differences in ocular anatomy and physiology can significantly affect the biomechanical behavior. For example, the strength of the neurosensory retina to RPE adhesion, measured by the difference in pressure across the retina in vivo, is approximately 1.4 and 1.8 times stronger in primates and cats, respectively, compared to rabbits [36]. Differences in ILM thickness across species can also impact overall retinal mechanics, as the ILM greatly contributes to the tensile strength of the retina [1, 17]. These interspecies differences have important implications for the interpretation of biomechanical results and for the translational potential. The macula, or fovea, concentrates tractional forces at the vitreoretinal interface and is central to clinically relevant conditions, such as vitreomacular traction and macular hole; its absence in rodents limits the translational validity of tractional adhesion data derived from these species. Furthermore, the porcine retina, which is vascularized and of similar overall thickness to the human retina, offers a closer structural homology and is, therefore, better suited for layer-specific mechanical characterization and surgical simulation. However, the porcine retina exhibits significant differences in viscoelastic properties compared to human tissue, and researchers should account for these mechanical discrepancies when extrapolating findings related to layer-specific mechanics or surgical tissue handling to the human clinical setting. Despite the limitations of animal models, human retinal tissue remains relatively underutilized in biomechanics research, possibly due to the scarcity of fresh donor eyes and challenging logistics.

### Mathematical and computational modeling

Mathematical and computational modeling is an evolving tool in retinal biomechanics, offering a framework to explore the mechanical environment of the eye and the subsequent effect of disease. Drawing on principles from solid and fluid mechanics, these models simulate the behavior of ocular tissues and the dynamics of intraocular fluids. They serve as complementary approaches in the characterization and prediction of retinal behavior, addressing questions that cannot be fully explained through biochemical and physiological methods alone [37]. Compared to traditional in vitro and in vivo approaches, mathematical and computational models offer several advantages: they can isolate specific mechanisms that are not feasible to test experimentally, and simulate a wide range of physiological and pathological scenarios without the constraints of time [37].

Geometrically, the retina is modeled differently depending on the phenomenon being investigated. To simulate retinal detachment, the retina is modeled as an elastic spherical shell supported by a rigid choroid–RPE–sclera base, with axisymmetric deformation and detachment [38–40]. The models of retinal detachment predict that once detachment ensues, it propagates in an unstable manner and that myopic eyes exhibit increased susceptibility to detachment, consistent with clinical findings [37, 39]. However, these models rely on material parameters drawn from the literature, such as a retinal Young's modulus of approximately 2 × 10^4^ Pa, and parametric studies demonstrate that predictions of detachment threshold and extent are sensitive to variations in retinal elastic modulus, retina–RPE bond energy, and the size of retinal tears [37–39]. For vitreoretinal traction models, Repetto et al. showed that saccade-induced tractions on the retina are of the same order of magnitude as the adhesive force between the retina and RPE (~ 0.1–1 Pa), providing indirect validation of the model [40].

In some situations, a two-dimensional model may be adequate, such as fluid–structure interaction and vitreoretinal traction, when the retina is a flat or spherical rigid domain [41–43]. To represent blood flow mechanics, the retina can be modeled as a three-dimensional, layered spherical structure with an embedded vascular network [44]. The governing equations for these models are rooted in continuum mechanics, differentiated by whether the retina and neighboring structures are treated as solids (e.g., elasticity, stress/strain, equilibrium) or fluids (e.g., Navier–Stokes, viscosity). When fluid and solid domains interact (e.g., the vitreous and the retina), coupling equations, including the Immersed Boundary method and the Arbitrary Lagrangian–Eulerian method are applied to study the fluid–structure interaction [41, 43, 44].

Overall, while these models demonstrate qualitative agreement with clinical observations, none have been fully validated against patient-specific quantitative biomechanical measurements. Mechanical models of the retina are often limited by simplifying assumptions, the degree of model complexity, and restricted biomechanical data. These gaps in experimental data require models to be based on assumptions, resulting in variability across models and potentially reducing confidence in predictive accuracy [37]. There is also a trade-off between model complexity and computational feasibility. For example, fluid–structure interaction models, incorporating fluid dynamics, can capture realistic retina behavior but are computationally intensive [44]; therefore, many resort to simplified elastic models. Furthermore, common modeling assumptions, such as treating the retina as a linearly elastic, isotropic material with ideal geometry [37], can oversimplify the retina’s complex non-linear, anisotropic and multi-layered structure.

## Cellular contributions to retinal biomechanics

### Role of Müller Glia

**Fig. 6:**
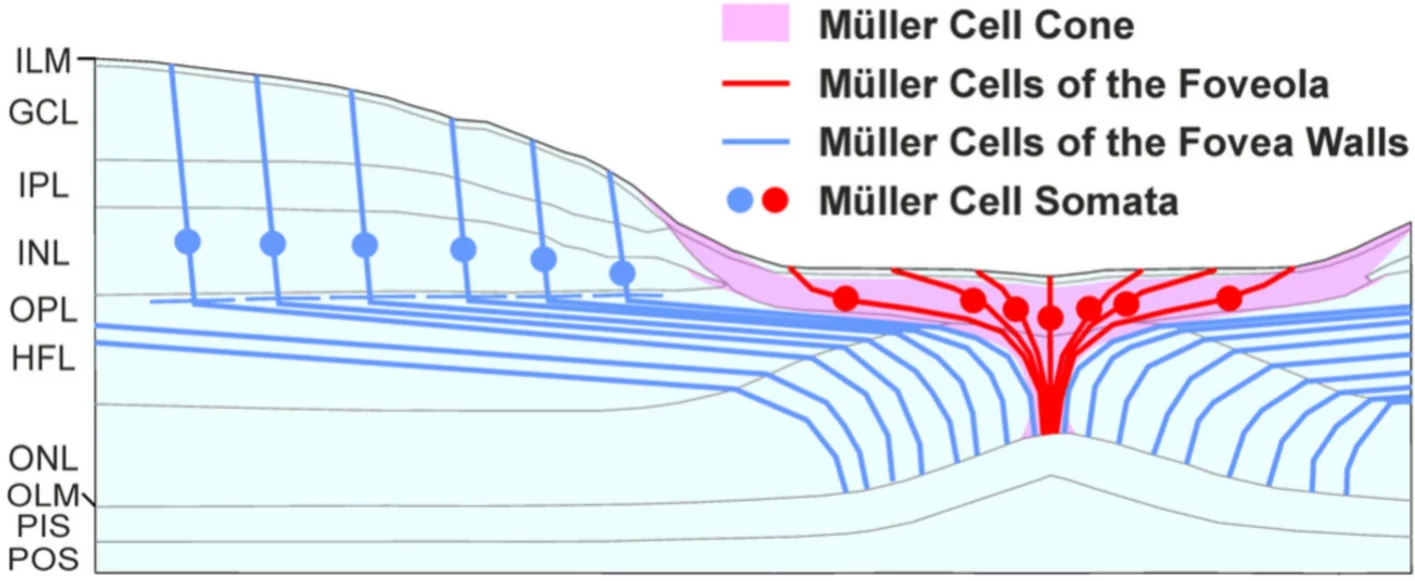
Organization of Müller cells in the fovea. The outer processes of foveolar Müller cells extend from the somata to the outer limiting membrane and inner processes line the basal surface of the internal limiting membrane, forming the Müller cell cone (pink). Müller cells of the foveal walls exhibit a z-shape due to displacement of inner and outer retinal layers. Figure adapted from Reichenbach and Bringmann [47]

Müller glial cells contribute to retinal biomechanics due to their unique morphology, intrinsic mechanical properties, mechanosensing abilities, and role in modulating the extracellular matrix. Müller cells span the entire neurosensory retina, with nuclei located in the inner nuclear layer and radial bipolar processes that extend across retinal layers, branching tangentially to contact neighboring neurons and blood vessels [45]. The substantial radial reach of Müller cells has been shown to provide tensile strength to the retina, acting akin to taut springs that help the tissue withstand deformation [46]. This is supported by developmental zebrafish studies, where Müller cells experience tension in the retina and in their absence, the retina becomes more susceptible to intraretinal tearing [46]. In the foveola, Müller cells form cone-like structures aiding light transmission and stability (Fig. 6) [47, 48]. Comparatively, in the parafoveal region, Müller cells adopt a Z-shaped geometry (Fig. 6) [47, 48], potentially protecting photoreceptors from tractional forces through an increased resistance to deformation [49]. This is supported in a mathematical study by Govetto et al., where Müller cells were modeled as solid structures with three segments (vertical segment from ILM to outer plexiform layer, horizontal segment in outer plexiform layer, and vertical segment from outer nuclear layer to outer limiting membrane) and two elastic joints of rotation [49]. Results found that the parafoveal Müller cells in their relaxed configuration, where the horizontal segment formed right angles with rotational joints, show a decrease in stiffness and transmit less tension to underlying photoreceptors.

Müller cells also possess unique intrinsic mechanical properties that align with their structural and mechanosensitive roles. Scanning force microscopy and tensile testing have demonstrated that Müller cells are viscoelastic, primarily behaving as elastic solids, with their elastic component (storage modulus) dominating over their viscous component (loss modulus) [50, 51]. In addition, the cell bodies of Müller cells exhibit lower elastic and viscous moduli than bipolar and amacrine neurons within the same retinal layers. This decreased stiffness may indicate that Müller cells provide a compliant environment for neurons, offering shock absorption in the event of mechanical trauma.

Beyond providing passive support, Müller cells are also mechanosensors and actively respond to mechanical stimuli. This mechanosensitive function is facilitated in part by the expression of stretch-activated ion channels, including the transient receptor potential vanilloid type 4 and the transient receptor potential canonical type 1, which are implicated in mediating calcium influx in response to mechanical stress [52]. Lindqvist et al. demonstrated that acute perpendicular stretch (at right angles to the tissue surface) of guinea pig retinas induced calcium increases in Müller cells [53]. This response was followed by the upregulation of signaling molecules, showcasing a transcriptional cascade induced via stretch. Extending this understanding, Jo et al. conducted radial tensile loading and identified the transient receptor potential vanilloid type 4 as the principal channel responsible for this calcium signaling, with the transient receptor potential canonical type 1 having a secondary role [52]. These channels trigger rapid calcium signaling that often initiates at the Müller endfeet and spreads along their radial processes.

Müller cells also exhibit cytoskeletal remodeling in response to mechanical stress, a hallmark of reactive gliosis [54]. This remodeling is characterized by an upregulation of intermediate filament proteins, including glial fibrillary acidic protein and vimentin, which influence cell stiffness and the composition of the retina’s extracellular matrix. Mechanical alterations, including changes in extracellular matrix stiffness, as seen in some retinal conditions, are sensed by mechanosensitive ion channels on Müller cells [55]. In addition to intracellular signaling, these channels can trigger a process of Müller cell proliferation and hypertrophy, referred to as gliosis [54, 55]. Initially, these reactive responses are neuroprotective, releasing antioxidants, neurotrophic factors, and increasing glutamate clearance [56]. However, excessive gliosis can become maladaptive, leading to a stiffening of reactive Müller cell endfeet and inner processes through increased intermediate filament expression [26, 54, 55]. This resulting increase in elastic modulus may contribute to glial scar formation, impeding the regeneration of neurons [54, 57].

### Photoreceptor and epithelium coupling

**Fig. 7:**
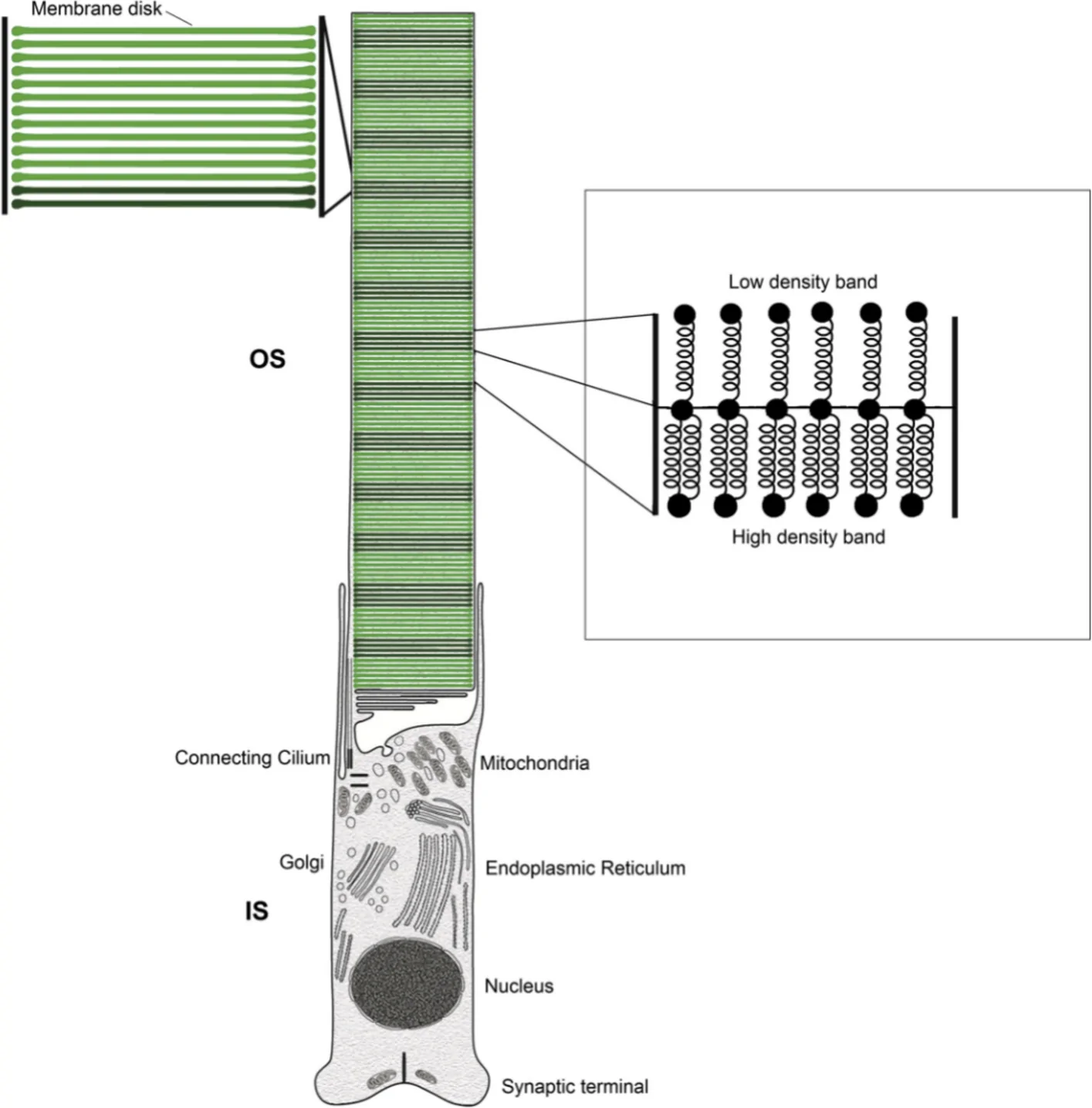
Diagram of a rod photoreceptor showing the inner (IS) and outer segment (OS), composed of stacked membrane disks grouped into alternating high-density (green) and low-density (black) bands. The high-density bands indicate stiffer regions prone to breakage, while the low-density bands have greater flexibility, together characterizing the overall bending stiffness of the outer segment. Figure adapted from Haeri et al. [58]

The photoreceptor is divided into an inner segment (IS) and an outer segment (OS), joined by a thin connecting cilium [58]. The rod OS is characterized by a cylindrical stack of enclosed disks distinct from the plasma membrane, whereas cones have a shorter tapered OS and disks are continuous with the plasma membrane [59, 60]. The rod OS is not axially uniform but shows alternating high- and low-density bands influenced by the light–dark cycle [58]. High-density bands, rich in rhodopsin and linked by protein spacers, provide local stiffness, while the low-density regions provide greater flexibility, both contributing to bending and fracture thresholds (Fig. 7) [58]. These features give the rod OS a bending stiffness, in the order of 10^5^ nN·μm^2^, calculated through mathematical modeling representing the OS as a spring-bead model [58]. Therefore, rods maintain a balance of rigidity and flexibility, as the OS must be stiff enough to resist deformation from stressors, including ocular movements or the renewal of OS disks, yet flexible enough to avoid fracture.

The interaction between photoreceptor OSs and the RPE is a mechanical relationship that stabilizes the retina. RPE cells extend apical microvilli that interact with outer segments, increasing contact area and forming a tight mechanical coupling without true anatomical junctions [61]. These RPE microvilli are supported internally by actin filaments and microtubules, which maintain the polarized characteristics of the outer retina [61]. In the healthy retina, this contact, along with the interphotoreceptor matrix, creates a strong adhesion between the neurosensory retina and the RPE. Experimental studies in rabbit models have shown that this adhesive force is approximately 100–180 dyn/cm [62].

An important aspect of the photoreceptor–RPE interface is the daily phagocytosis of shed OS tips, required to mitigate oxidative stress from prolonged light exposure [63]. RPE cells engulf the distal portion of the rod and cone OS, a process that generates mechanical loads on both cell types [61]. During this phagocytosis, the RPE’s actin–myosin cytoskeleton actively remodels to internalize fragments [61]. Korpela et al. showed that this process produces a measurable traction with RPE cells exerting lateral traction stresses of approximately 430 ± 180 Pa on the apical surface during outer segment engulfment [64]. This demonstrates that the RPE applies active pulling forces on photoreceptor tips during phagocytosis.

## Extracellular contributions to retinal biomechanics

In addition to its cellular composition, the retina is supported by an acellular framework. The ECM plays a role in maintaining tissue structure and lamination, facilitating intercellular communication, and regulating biomechanical and biochemical homeostasis [8]. The retinal ECM acts as a composite material containing fibrous proteins (collagen, elastin), hydrated polymers (proteoglycans and glycosaminoglycans), and glycoproteins (fibronectin, laminin) [1]. This scaffold provides the retina with non-linear, anisotropic, and viscoelastic properties, allowing it to withstand intraocular pressure, ocular motion, and tractional forces without structural failure [8, 22].

The retinal ECM is distributed across several compartments, including the basement membranes (i.e., the ILM and vascular basement membranes), and the interstitial matrix distributed across the retina between the ILM and the RPE [65, 66]. At the vitreoretinal interface, the ILM forms a collagen IV-based basement membrane that functions as a rigid sheet anchoring Müller glial endfeet and reinforcing vitreoretinal adhesion [22, 32]. Within the neurosensory retina, the interstitial matrix fills the nerve fiber layer, plexiform layers and the interphotoreceptor matrix, composed largely of proteoglycans and glycoproteins that regulate shear resistance and guide synaptic organization [8]. Finally, in the subretinal space, the interphotoreceptor matrix surrounds the inner and outer photoreceptor segments, adhering photoreceptors to the RPE [66].

### Collagens

**Fig. 8:**
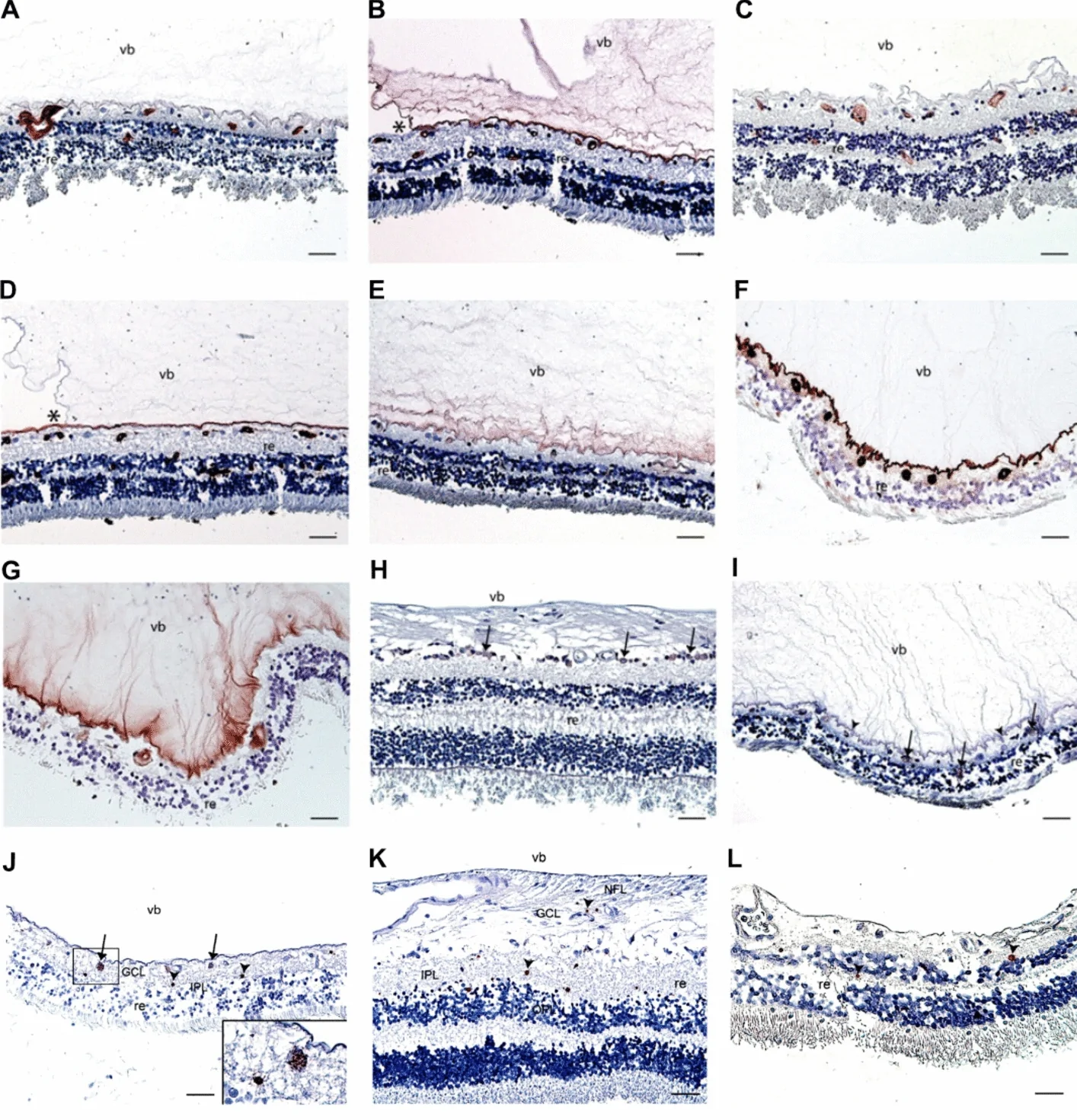
Distribution of various collagen types in the human retina (re) and vitreous body (vb), identified through immunohistochemical staining (red/brown indicates positive labeling). **A**–**I** Collagens type I–VI, IX, XI, and XVII. **A**) Type I, **B** Type II, **C** Type III, **D** Type IV, **E** Type V, **F** Type VI, **G** Type IX and I Type XVIII show positive labeling in the retinal vasculature. **D**, **F**, and **I** exhibit additional staining along the internal limiting membrane. Positive vitreous staining is observed for **B** Type II, **E** Type V, and **G** Type IX. **H** Type XI shows localized deposits in the ganglion cell layer. **J**–**L** Type VII collagen distribution. Positive labeling found within the ganglion cell layer (**J**, **K**), the nerve fiber layer (**K**), the inner plexiform layer (J, K), the outer plexiform layer (**K**, **L**), and the outer nuclear layer (**L**). Scale bars = 50 µm. Figure adapted from Ponsioen et al. [69]

Collagens are the primary load-bearing fibers within the retinal ECM, providing tensile strength, with different isoforms (I–VII and XVIII) distributed across the retinal layers (Fig. 8) [8, 67]. Collagen type I is localized to the ganglion cell layer and the ILM, where it mediates adhesion and is upregulated during glial scarring following retinal injury [8, 68, 69]. Type II collagen, a major vitreous component, extends into the ILM and forms dense fibrillar bundles within the nerve fiber layer and IPL, anchoring the vitreous cortex to the retina [69, 70]. Type IV collagen accounts for approximately 60% of ILM protein content [23] and interacts with laminin and fibronectin to form a cross-linked basement membrane meshwork that supports Müller glial endfeet and RGCs [68, 70]. Collagen VI filaments interlink with collagen IV, anchoring vitreous fibrils and enhancing vitreoretinal adhesion [68]. Collagen VII deposits are observed in the inner nuclear layer, IPL and outer nuclear layer, suggesting a region-specific role in adhesion (Fig. 8) [69, 70]. Finally, collagen XVIII contributes to vascular stability and contains endostatin, an anti-angiogenic fragment critical for retinal homeostasis in vascular basement membranes [67].

### Extracellular space

Quantitative measurements of extracellular space (ECS) volume fraction provide additional information on the stiffness gradient seen in Brillouin microscopy and OCE. Using two-photon calcein fluorescence imaging, Kuo et al. demonstrated that synaptic plexiform layers exhibit substantially higher ECS volume fractions, ranging from 0.087 ± 0.009 in the outer plexiform layer to 0.122 ± 0.009 in the IPL, compared to ECS volume fractions of 0.026 ± 0.03 in the outer nuclear layer and 0.025 ± 0.03 in the inner nuclear layer [71]. These low ECS fractions in nuclear layers indicate dense cellular packing, which may increase mechanical stiffness. In contrast, the higher ECS fractions in plexiform layers reflect a more porous organization, with greater interstitial fluid volume and less densely packed cells, potentially resulting in decreased mechanical stiffness.

In addition to baseline structural differences between retinal layers, synaptic activity may influence local mechanical properties through the regulation of ECS volume and cellular swelling. Rapid reductions in the ECS volume fraction have been observed following light stimulation in the adult mouse retina, with the greatest decrease occurring in the IPL (~ 10%) [71]. These changes are thought to result from neuronal and/or glial swelling, driven by ion fluxes associated with synaptic transmission, including potassium accumulation in the ECS and subsequent influx of sodium and bicarbonate ions into Müller cells [71]. Such reductions in ECS volume fraction may lead to transient increases in local stiffness through cytoskeleton reorganization.

## Clinical and pathological relevance of retinal biomechanics

### Myopia

Myopia, commonly known as nearsightedness, is a refractive error in which light is focused in front of the retina, causing distorted distance vision [72]. Globally, the prevalence is estimated to be 30%, with projections suggesting it will rise to 40% by 2050 [73]. In cases of axial myopia, the eye undergoes axial elongation, resulting in stretching of and mechanical stress on the retina [74]. Ito et al. suggest that myopia induces remodeling of choroidal stroma and sclera, where choroidal stroma in the peripheral region becomes less dense and central areas stiffen [75]. This sustained mechanical load results in retinal thinning [74], more pronounced in the inner retinal layers, nerve fiber layer and IPL, compared to outer layers [76–78]. Further work is required to determine if a similar change happens on the retinal ECM as that of the sclera [74].

Mechanically, an increase in axial length predisposes the eye to several pathologies, including myopic maculopathy [79], posterior vitreous detachment [80], and retinal detachment [75]. The link between myopia and retinal detachment is particularly significant, as epidemiological studies show that higher myopic refractive error and longer axial length correlate with greater risk of rhegmatogenous retinal detachment [81]. An energy-based model of retinal detachment, incorporating retinal biological growth (elongation) and elastic deformation imposed by an expanding globe myopia, revealed that the critical stress required for the propagation of detachment is lower in the myopic retina compared to emmetropic, and that at high levels of myopia there is sufficient energy release for the propagation of detachment, even in the absence of an applied stress [40]. These results provide insight into the mechanical causation—a chronically stretched, and possibly thinner, retina is less resilient to detaching forces.

### Posterior vitreous detachment

Posterior vitreous detachment is the biomechanical process in which the posterior vitreous cortex separates from the retina, typically in response to vitreous liquefaction and weakening of vitreoretinal adhesion [42]. As the vitreous ages, it undergoes liquefaction and contraction, processes that alter the tissue from a single-phase viscoelastic solid into a composite of gel and fluid with variable stiffness. These changes reduce dampening and interrupt mechanical homeostasis in the vitreous body, increasing its mobility and susceptibility to deformation under external forces, such as eye movements. Repetto et al. demonstrated that these changes contribute to non-uniform mechanical loading on the retina, which may increase the risk of traction-induced damage [41].

Mechanical modeling of posterior vitreous detachment offers insight into how regional differences in vitreoretinal adhesion influence retinal stress. Di Michele et al. developed a continuum model of posterior vitreous detachment that treated the vitreous gel as a shrinking elastic body surrounded by an elastic membrane within a rigid spherical eye [42]. In their model, the strength of vitreoretinal adhesion was assumed to be spatially non-uniform, typically weakest at the posterior pole and increasing toward the vitreous base. As the vitreous shrinks and detaches, this variability in adhesion creates uneven, localized tractions on the retina. Furthermore, in cases of atypical posterior vitreous detachment with vitreomacular adhesion, the macula was identified as the region subjected to the highest traction, which can lead to macular hole formation.

### Retinal detachment

Retinal detachment is an ocular condition involving the separation of the neurosensory layers of the retina from the underlying RPE, accompanied by an infiltration of subretinal fluid. Cases of posterior vitreous detachment can transition to retinal detachment when mechanical forces exceed the structural integrity of the retinal tissue, leading to a retinal tear [42]. Retinal detachment involves dynamic interactions between vitreous contraction, subretinal pressure, and the mechanical properties of the retina. Vitreous contraction and the stretching of vitreous fibrils generate localized stress and interfacial pressure at adhesion points, which can initiate a tear [39]. Once a break occurs, liquified vitreous enters the subretinal space, where the resulting fluid pressure separates the neurosensory retina from the underlying RPE [42]. Mechanically, these events span different timescales. Gradual vitreous degeneration imposes slow, quasi-static stresses on the retina over years, while the final detachment often unfolds as a rapid, dynamic event [39].

Mathematical models of retinal detachment propagation help illustrate these phenomena. An analytical energy-based model for the detaching retina, treating the retina as a spherical elastic shell on an RPE foundation and considering vitreous contraction, vitreous fibril tension, and subretinal pressure as driving forces, was used to predict that once detachment is initiated, it propagates catastrophically, where detachment enlarges without additional increases in stress [38]. Interestingly, it was also found that the presence of a pre-existing retinal tear can have a stabilizing effect on the spread of detachment, providing an outlet for stress relief and effectively limiting how far the detachment can propagate. Lakawicz et al. improved upon this model through the inclusion of meridional, out of plane, displacements of the retina. Incorporating this extra degree of freedom resulted in substantially larger retinal displacements and lowered the predicted critical stress necessary for detachment [39]. In other words, when the retina deforms out of plane, even less force is required to continue detachment, suggesting that previous models underestimated how easily an initiated detachment could progress. Despite this change, the redefined model still held the prediction of abrupt propagation and tear stabilization.

Surgical repair of retinal detachment also has important biomechanical considerations, as it aims to restore retinal apposition and relieve traction [82, 83] through approaches, such as scleral buckling, pars plana vitrectomy and pneumatic retinopexy [82]. These interventions modify the eye’s mechanical environment, including ocular rigidity and vitreoretinal interface dynamics [83], and must relieve both the immediate detachment and the underlying tractional stresses to prevent recurrence.

### Diabetic retinopathy

In diabetic retinopathy, a vision-threatening complication of diabetes mellitus, chronically high blood glucose levels cause damage to the retinal vasculature and alter the biomechanical properties. One defining feature of long-term diabetes is a thickening and stiffening of the capillary basement membranes and the ILM [84, 85]. Ultrastructural studies showed that after a decade or more of diabetes, retinal capillary basement membranes have an increased thickness of approximately 2.5 μm compared to non-diabetic eyes [85]. Similarly, the ILM in diabetic eyes is both thicker and mechanically stiffer than in healthy eyes. AFM measurements have shown that diabetic ILMs have an average elastic modulus of 37.9 kPa, compared to 24 kPa in age-matched controls, with a 20–60% increase in stiffness.

Retinal stiffening is linked to chronic hyperglycemia due to glycation and protein accumulation [86]. Concurrently, hyperglycemia upregulates the production of key ECM components in the retinal vasculature, most notably collagen IV and fibronectin, resulting in their abnormal accumulation in capillary walls and the ILM [85]. These biochemical alterations are compounded by hemodynamic changes in diabetic retinopathy. For instance, elevated retinal venous pressure in diabetic eyes can induce retinal hypoxia and edema, which in turn activates mechanosensitive ion channels on endothelial cells [87]. The activation of such channels increases blood–retinal barrier permeability and promotes fluid leakage into retinal tissue, further disrupting homeostasis [87]. The net effect of these changes is a stiffer, less adaptable retina. Importantly, the biomechanical alterations impair the retina’s ability to withstand tractional forces and mechanical stress. In advanced diabetic retinopathy, a rigid, thickened ILM and vasculature make the retina less pliable, which can complicate vitreoretinal surgical interventions, such as vitrectomy, and reduce the tissue’s capacity to recover from mechanical strain.

Computational models provide further insight into how diabetes-related stiffening may affect retinal biomechanics and hemodynamics. Using a fluid–structure interaction model, Rebhan et al. simulated blood flow in three-dimensional, six-layer retinal reconstructions derived from human fundus images representing healthy, glaucomatous and diabetic eyes [44]. By varying flow conditions (steady vs. pulsatile), viscosity models (Newtonian vs. non-Newtonian), and tissue properties (rigid vs. compliant), they computed wall shear and von Mises stresses. Findings indicated that retinal mechanical properties can directly influence vascular flow dynamics, with wall shear stress in retinal arterioles found to be sensitive to the compliance of the surrounding retina. The simulated diabetic retina, characterized by altered geometry and stiffened tissue, exhibited elevated wall shear stress compared to healthy controls. This evidence suggests that diabetes-induced retinal stiffening actively modifies the mechanical forces experienced by retinal blood vessels, potentially influencing disease progression by altering vascular mechanosensitivity.

## Conclusions and future directions

Retinal biomechanics is an emerging interdisciplinary field that bridges engineering research with clinical objectives. Knowledge gained from experimental studies and computational analyses shows the importance of incorporating mechanical properties into ocular models; however, future research in retinal biomechanics must address variability and data limitations that currently challenge the field. Experimental measurements of retinal mechanical properties often differ across studies due to variations in sample preparation, measurement methods, and species characteristics. Development of novel techniques and modeling for multi-layered tissue, such as the retina, is essential for the mechanical characterization and comparison of study results. In addition, the limited use of human donor tissue restricts the clinical relevance of retinal mechanics, highlighting the need for mechanical characterization studies on human retinal tissue.

Advancing non-invasive imaging and measurement technologies represents another important direction for future research. Novel methodologies, such as optical coherence elastography, show promise for quantifying mechanical stiffness and deformation in vivo, connecting experimental data with the native physiological ocular environment. These tools can facilitate mechanical mapping across different retinal regions and layers, capturing spatial heterogeneity and tissue properties, providing more physiologically relevant simulations of retinal responses to surgical manipulation, injection-induced deformation, and disease-related remodeling. Furthermore, longitudinal non-invasive testing techniques will be especially important for understanding how retinal mechanics evolve with age or disease progression.

The translation of retinal biomechanics into clinical practice is increasingly relevant and offers promising opportunities. Intravitreal and subretinal injections are an increasingly common method of administering therapies to the retina, and retinal biomechanics could play a role in efficacy and complications. For example, finite element models of injection-induced retinal deformation could help predict safe injection parameters and minimize tissue damage. Likewise, advances in robotic microsurgery can utilize biomechanical principles to improve surgical planning and handling of retinal tissue, reducing the risk of damage. Finally, utilizing patient-specific biomechanical models could inform personalized treatment strategies, allowing clinicians to tailor interventions based on individual tissue properties. Future work in standardizing methods, leveraging imaging technology, and improving models for clinical translation will be important in furthering our understanding of the retina’s mechanical environment in health and disease.

## Supplementary Information

Supplementary file1 (DOCX 26 KB)

## Data Availability

No datasets were generated or analyzed during the current study.

## Abbreviations

AFM: Atomic force microscopy
ECM: Extracellular matrix
ECS: Extracellular space
GCL: Ganglion cell layer
ILM: Internal limiting membrane
IOP: Intraocular pressure
IPL: Inner plexiform layer
IS: Inner segment
OCE: Optical coherence elastography
OCT: Optical coherence tomography
ONL: Outer nuclear layer
OPL: Outer plexiform layer
OS: Outer segment
RGC: Retinal ganglion cell
RPE: Retinal pigment epithelium
