# Supplementary material for: Biomechanics of the retina: experimental and clinical perspectives
Source: Biomed Eng Online. 2026 Jul 7;25:109. doi: 10.1186/s12938-026-01599-2 (PMC13625495; doi:10.1186/s12938-026-01599-2)
Supplement: Supplementary file 1 [file 12938_2026_1599_MOESM1_ESM.docx]

**Supplementary Table 1.** Summary of uniaxial tensile testing parameters and reported elastic moduli values for retinal tissue. Variations in tissue characteristics (species, location, orientation, vasculature) and testing conditions (strain rate, hydration state, temperature) are summarized to illustrate sources of mechanical variability across studies.

| **Tissue Characteristics** | **Testing Conditions** | **Elastic Moduli** | **Reference** |
| --- | --- | --- | --- |
| • Species: Porcine  • Location: Center of macula  • Orientation: Horizontal  • Vasculature: Major blood vessels avoided | • Strain Rate: 0.03mm/s, 1.65mm/s  • Hydration State: Not provided  • Temperature: Not provided | • Young's Modulus: 100kPa (0.03mm/s), 110kPa (1.65mm/s) | Wollensak & Spoerl, 2004 |
| • Species: Porcine  • Location: Posterior pole  • Orientation: Horizontal and vertical  • Vasculature: Blood vessels included | • Strain Rate: 1mm/s  • Hydration State: Air and saline  • Temperature: 25°C and 37°C | Toe Modulus  • Saline 25°C: 2.0±1.7 kPa (vertical), 0.8±0.9 kPa (horizontal)  • Saline 37°C: 0.7±0.5 kPa (vertical), 0.3±0.3 kPa (horizontal)   Heel Modulus  • Air 25°C: 82.4±73.8 kPa (vertical), 62.7±48.6 kPa (horizontal)  • Saline 25°C:107.8±58 kPa (vertical), 73.2±69.8 kPa (horizontal)  • Saline 37°C: 39.2±18.8 kPa (vertical), 19.5±12.2 kPa (horizontal) | Chen, Rowley & Weiland, 2010 |
| • Species: Porcine  • Location: Adjacent to optic nerve  • Orientation: Axial and circumferential  • Vasculature: Samples with superior-temporal vein/artery and no visible vessels | • Strain Velocity: 1mm/s  • Hydration State: Saline  • Temperature: 37°C | Transition Modulus  • Vein: 50.90±42.39 kPa, 47.58±27.16 kPa (axial), 24.67±11.88 kPa (circumferential)  • Artery: 38.48±16.64 kPa  • No visible vessel: 11.12±6.10 kPa | Chen & Weiland, 2010 |
| • Species: Porcine  • Location: Adjacent to optic nerve, near superior-temporal vessels  • Orientation: Not provided  • Vasculature: No visible vessels | • Strain Rate: 1mm/s  • Hydration State: Saline  • Temperature: 37°C, 26.1°C, 7.8°C | Transition Modulus  • Saline 37°C: 11.12±6.10 kPa (Chen & Weiland, 2010) *  • Saline 26.1°C: 111.25±88.16 kPa  • Saline 7.8°C: 125.13±63.61 kPa | Chen & Weiland, 2012 |
| • Species: Human  • Location: Posterior pole  • Orientation: Horizontal and vertical  • Vasculature: Blood vessels included | • Strain Rate: 1mm/s  • Hydration State: Saline  • Temperature: 37°C | • Toe Modulus: 0.3±0.6 kPa (vertical), 0.2±0.2 kPa (horizontal)  • Heel Modulus: 18.6±16.3 kPa (vertical), 12.5±10.1 kPa (horizontal) | Chen et al., 2014 |
| • Species: Human  • Location: Posterior pole  • Orientation: Not provided  • Vasculature: Superior and inferior arteries | • Strain Rate: 1mm/s  • Hydration State: Saline  • Temperature: 37°C | • Toe Modulus: 0.91±0.79 kPa  • Heel Modulus: 30.52±17.68 kPa | Chen & Weiland, 2014 |
| • Species: Human and porcine  • Location: Center of macula  • Orientation: Horizontal and vertical  • Vasculature: Blood vessels included | • Strain Rate: 50%/s  • Hydration State: Saline  • Temperature: 37°C | Linear Elastic Response  • Human: 122.526 kPa (horizontal), 39.149 kPa (vertical)  • Porcine: 1.362±0.118 kPa (horizontal), 1.454±0.335 kPa (vertical) | Zhang et al., 2018 |
| • Species: Porcine  • Location: Not provided  • Orientation: Not provided  • Vasculature: Blood vessels included | • Strain Rate: 80%/s  • Hydration State: Saline  • Temperature: Room temperature | • Elastic Modulus: 2870±1290 kPa | Belgio et al., 2020 |
| • Species: Porcine  • Location: Center of retina  • Orientation: Equatorial  • Vasculature: Blood vessels included | • Strain Rate: 0.1mm/s  • Hydration State: Air  • Temperature: Room temperature | • Elastic Modulus: 13.4±0.0067 kPa | Belgio, Boschetti & Mantero, 2021 |
| • Species: Porcine  • Location: Not provided  • Orientation: Not provided  • Vasculature: No visible vessels | • Strain Rate: 0.1mm/s  • Hydration State: Air, saline  • Temperature: 25°C, 37°C | Elastic Modulus  • Air 25°C: 6±1.7 kPa  • Saline 25°C: 7.2±1.9 kPa  • Saline 37°C: 8.65±4.01 kPa | Belgio et al., 2025 |

**Supplementary Table 2.** Comparative table to summarize strengths and limitations of each technique for biomechanical testing of the retina.

| **Technique** | **Spatial Resolution** | **Invasiveness** | **Measurable Parameters** | **In Vivo Capabilities** | **Translational Readiness** | **Key Limitations** |
| --- | --- | --- | --- | --- | --- | --- |
| Uniaxial / biaxial tensile testing | Low Bulk tissue (mm scale) | Highly invasive | Young's modulus, ultimate tensile strength, stress relaxation, creep, viscoelasticity, anisotropy | No | Low | Ex vivo only; tissue preparation alters properties; cannot resolve layer-specific mechanics |
| Atomic force microscopy | High nm – μm | Invasive | Young's modulus, local stiffness, viscoelasticity, surface topography | No | Low | Ex vivo; surface/near-surface only; Hertz model assumptions invalid for soft viscoelastic tissue; sensitive to probe calibration and indentation rate |
| Brillouin microscopy | High ~1 μm (subcellular) | Non-contact | Longitudinal modulus, viscoelastic properties, spatially resolved stiffness maps | Limited | Emerging | GHz-frequency modulus not directly comparable to quasi-static moduli; requires knowledge of local density and refractive index; slow acquisition; photo safety constraints in vivo |
| Optical coherence elastography | Moderate μm – mm | Minimally invasive | Young's modulus, shear modulus, wave speed, depth-resolved stiffness maps | Yes | Moderate | Modulus extraction requires simplifying assumptions (isotropy, homogeneity); sensitive to excitation frequency and amplitude; motion artefacts in vivo; may not resolve individual sublayers |
